# Soft Tissue/Bone Decomposition of Conventional Chest Radiographs Using Nonparametric Image Priors

**DOI:** 10.1155/2019/9806464

**Published:** 2019-06-24

**Authors:** Yunbi Liu, Wei Yang, Guangnan She, Liming Zhong, Zhaoqiang Yun, Yang Chen, Ni Zhang, Liwei Hao, Zhentai Lu, Qianjin Feng, Wufan Chen

**Affiliations:** ^1^School of Biomedical Engineering and Guangdong Provincial Key Laboratory of Medical Image Processing, Southern Medical University, Guangzhou, Guangdong 510515, China; ^2^Laboratory of Image Science and Technology, Southeast University, Nanjing 211189, China; ^3^Key Laboratory of Computer Network and Information Integration, Southeast University, Ministry of Education, Nanjing 210096, China; ^4^Nanfang Hospital, Southern Medical University, Guangzhou 510515, China

## Abstract

**Background and Objective:**

When radiologists diagnose lung diseases in chest radiography, they can miss some lung nodules overlapped with ribs or clavicles. Dual-energy subtraction (DES) imaging performs well because it can produce soft tissue images, in which the bone components in chest radiography were almost suppressed but the visibility of nodules and lung vessels was still maintained. However, most routinely available X-ray machines do not possess the DES function. Thus, we presented a data-driven decomposition model to perform virtual DES function for decomposing a single conventional chest radiograph into soft tissue and bone images.

**Methods:**

For a given chest radiograph, similar chest radiographs with corresponding DES soft tissue and bone images are selected from the training database as exemplars for decomposition. The corresponding fields between the observed chest radiograph and the exemplars are solved by a hierarchically dense matching algorithm. Then, nonparametric priors of soft tissue and bone components are constructed by sampling image patches from the selected soft tissue and bone images according to the corresponding fields. Finally, these nonparametric priors are integrated into our decomposition model, the energy function of which is efficiently optimized by an iteratively reweighted least-squares scheme (IRLS).

**Results:**

The decomposition method is evaluated on a data set of posterior-anterior DES radiography (503 cases), as well as on the JSRT data set. The proposed method can produce soft tissue and bone images similar to those produced by the actual DES system.

**Conclusions:**

The proposed method can markedly reduce the visibility of bony structures in chest radiographs and shows potential to enhance diagnosis.

## 1. Introduction

Chest radiography is a widely used diagnostic imaging technique for lung diseases, such as tuberculosis, pneumonia, and lung cancer, because this method is cheap, routinely available, and relatively safe. However, overlying anatomical structures, such as ribs and clavicles, make the reading and interpretation of chest radiographs difficult for radiologists. Such inaccurate analysis may cause serious decision-making errors. Studies showed that approximately 30% of pulmonary nodules in chest radiographs could be missed by radiologists, and 82% to 95% of such missed nodules are partly obscured by ribs and clavicles [1]. Therefore, suppression of ribs and clavicles in chest radiographs would be potentially useful for improving the detection accuracy of radiologists.

**Figure 1:**
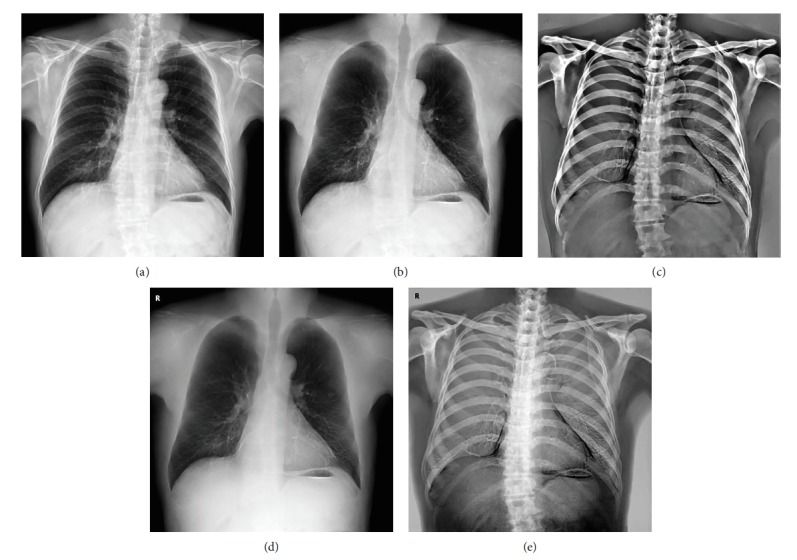
An example of the processed DES training set: (a) the standard chest radiograph; (b) the processed DES soft tissue image; (c) the processed DES bone image; (d) the soft tissue image directly obtained by the DES system; (e) the bone image directly obtained by the DES system.

One method to reduce the visual clutter of chest radiographs from overlying anatomy is DES imaging [2]. DES radiography involves capturing two radiographs with the use of two X-ray exposures at two different energy levels. These radiographs are then combined to form a subtraction image that highlights either soft tissue or bone components, as shown in Figure 1. The soft tissue image can achieve improved visualization of pulmonary nodules because the ribs and clavicles become invisible in this approach. DES chest radiography exhibits many advantages over conventional chest radiography in terms of facilitating image interpretation. However, only a few hospitals use the DES system because of the required specialized equipment.

Another method to remove or suppress the bone components in chest radiographs is the image processing technique that does not require specialized equipment for DES. The commercial software ClearRead Bone Suppress (formerly SoftView) of Riverain Technologies is such a tool for bone suppression in chest radiographs. An early version of the MTANN (massive training artificial neural network) model for rib suppression proposed by Suzuki et al. [3] was evaluated on 60 chest radiographs. Oda et al. [4] found that a combination of rib-suppressed and original chest radiographs could significantly improve the diagnostic performance of radiologists over the use of chest radiographs alone for the detection of small pulmonary nodules. Suppression of bony structures in chest radiographs by using the image processing technique can improve radiologist performance in terms of nodule detection [5], as well as the performance of computer-aided nodule detection (CAD) [6]. Previous methods for bone suppression can generally be divided into two categories: supervised and unsupervised methods. The supervised methods treat bone suppression in chest radiographs as a regression prediction problem, and the regressors are trained or optimized by a DES training data set to estimate the soft tissue or bone images [3, 7, 8]. The soft tissue images are then reconstructed using the outputs of the regressor with the local image features as the direct inputs or by subtracting the outputs of the regressor from the chest radiographs based on the prediction target of the regressor. However, in the supervised methods, only local features and information of the input chest radiographs can be used to predict the soft tissue or bone images. The unsupervised methods for bone suppression do not require the training set, but these methods need segmentation and border locations of the bony structures as intermediate results [9, 10]. The bone-free images are reconstructed by the blind source separation approach or from the gradient images modified according to the intermediate results. The effectiveness of unsupervised methods highly depends on the accuracy of segmentation and border locations of bony structures.

Unlike the previous methods for the bone suppression of chest radiographs, we proposed a supervised method by treating the separation of soft tissue components from bone components as an image decomposition problem. We attempted to use the information of whole chest radiographs and the prior knowledge contained in the data set of real DES radiographs to infer the soft tissue and bone images. The decomposition of a single image is highly ill-posed, and the effective prior or regularization is a critical factor for obtaining the reasonable decomposed components. The general image priors, such as smoothness [11] and sparsity [12, 13], are too general to impose effective constraint on the solutions of soft tissue and bone images. Considering the visual characteristics of soft tissue and bone images, these images are distinctly different from the images of other anatomical positions or modalities. Soft tissue images are also distinctly different from bone images, as well as from natural texture images. Thus, we needed to construct specific priors of soft tissue and bone images for the decomposition. Inspired by the work of Tappen and Liu [14], we constructed nonparametric priors of soft tissue and bone images in the kernel density estimation framework. These nonparametric priors are then integrated into a Bayesian maximum a posteriori (MAP) model to estimate the soft tissue and bone images for a given chest radiograph.

The key issue in estimating the nonparametric probability density is sample selection. Given the assumption that if the local features of the patches at the close anatomical position in the chest radiographs are similar, the corresponding patches in the soft tissue and bone images should also be similar. We can search the nearest neighbors of unknown patches in the soft tissue and bone images according to the similarities among patches in chest radiographs. A simple way for sampling is to build a large data set of patch triplets (patches at the same spatial location of chest radiograph, soft tissue image, and bone image) and then search the nearest neighbors of each patch in a given chest radiograph to select the corresponding patches of the soft tissue and bone as samples for density estimation. The size of the data set of patch triplets should be large enough for accurate density estimation. However, a very large data set would lead to a huge computation cost of the nearest neighbor search for each patch, and the information of spatial layout about chest radiographs would be completely ignored. A more efficient way to find the nearest neighbors of patches between two images is the dense matching algorithms, such as SIFT Flow [15], PatchMatch [13], and deformable spatial pyramid (DSP) matching [16]. The corresponding relationship of similar patches between two images can be represented by the dense corresponding field or nearest-neighbor field. The dense matching algorithms can use the spatial smoothness prior of the corresponding fields to accelerate the search of the nearest neighbors of image patches. The smoothness of the corresponding fields can be ensured implicitly or explicitly, which is important to obtain more reasonable matching of patches. In the current study, a hierarchically dense matching algorithm is proposed to solve the corresponding fields by integrating DSP and PatchMatch algorithms.

Given a large data set of DES radiographs, another issue may occur regarding the selection of an effective subset of images as exemplars to estimate the priors. Matching a given chest radiograph to all chest radiographs in the training set would be time-consuming. To alleviate this problem, we selected several of the most similar images of the given chest radiograph as exemplars. Similarities among the images are defined based on their bag-of-words (BoW) histograms for rapid search and selection. Other issues of our decomposition method, such as the normalization of chest radiographs and optimization of decomposition energy function, are also addressed. Our method can produce decomposition results similar to those produced by the real DES system.

## 2. Methods

### 2.1. Image Data

The image data used in this study were collected from two data sets. The first data set consisted of 503 posterior-anterior DES chest radiographies acquired with a DES system (Revolution XR/d, GE) at Nanfang Hospital, Guangzhou, China. The X-ray tube voltages for the two exposures were 120 and 60 kV. The sizes of the chest radiographs ranged from 2011 × 2011 pixels to 2048 × 2048 pixels, and the pixel sizes ranged from 0.191 mm to 0.195 mm. The images were stored in a DICOM format with a 16-bit depth. The second one was the publicly available Japanese Society of Radiological Technology (JSRT) data set. The JSRT data set consisted of 247 standard posterior-anterior chest radiographic images, among which 154 images contained one pulmonary lung nodule, while the remaining 93 images contained no lung nodules. The nodule diameters range from 5 to 60 mm, and their intensities vary from nearly invisible to very bright. All the images were scanned from plain film radiographs (size: 2048 × 2048 pixels, pixel size: 0.175 mm).

We have collected 503 cases of DES chest radiographs from the first data set. 403 cases were randomly selected to construct the training set, and the remaining 100 cases were considered the validation set. Each of the DES image triplets in our collected data set included a standard CXR (denoted by **Y**), a DES soft tissue image (denoted by **S**^0^), and a DES bone image (denoted by **B**^0^). Given the sophisticated nonlinear postprocessing of the raw image data, the relationship **Y** = **S**^0^ + **B**^0^ was not eventually satisfied. To build our decomposition model, we need to process the DES images for exacting the bone component **B** and soft tissue component **S** to satisfy **Y** = **S** + **B**. The gradient **G** of the bone components in **Y** were obtained as the transformed gradient field of **Y** using cross projection tensors [17] from **B**^0^. The bone component **B** in **Y** was ultimately reconstructed from **G** through 2D integration. The corresponding soft tissue component **S** can be obtained as **Y**‐**B**. Finally, we constructed a training set containing the standard DES CXRs, the processed DES soft tissue, and bone images. The spatial resolution of images was then rescaled by the factors 0.25 and 0.3 and cropped by a rectangle centered at the images with a size of 512 × 512 pixels. An example of the processed training set is shown in Figure 1. In Figure 1, you can see that the contrast was enhanced in the processed DES soft tissue image compared to the original. The bony component can be seen more clearly in the processed DES bone image than the original. For convenience, the processed DES soft tissue and bone images are considered the DES images in the following.

### 2.2. Overview of the Proposed Method

**Figure 2:**
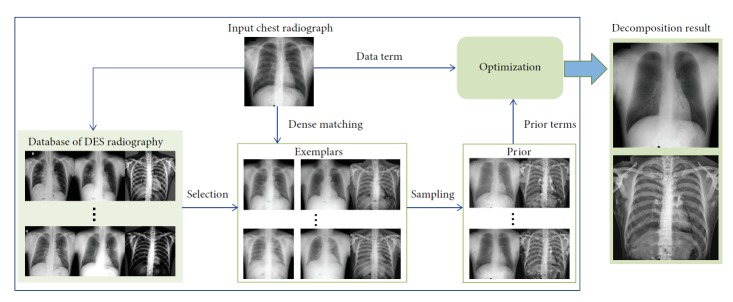
Flowchart of the proposed method for decomposition of a chest radiograph.

Figure 2 illustrates an overview of the decomposition of a standard chest radiograph using the proposed method. A database of the image triplets (chest radiographs and corresponding soft tissue and bone images) of DES radiographs has been established in advance. Given a standard chest radiograph as the input, its soft tissue and bone components are then produced with the following basic steps:

1. Search and find the exemplars from the database of DES radiographs according to the similarity between the representations of the input chest radiograph and the chest radiographs in the database
2. Solve the dense corresponding fields between the input image and the chest radiographs of exemplars using a dense matching algorithm
3. Construct the exemplar-based nonparametric priors for unknown soft tissue and bone images
4. Optimize an energy function and infer the soft tissue and bone components under a Bayesian MAP framework

The framework for the decomposition of a chest radiograph is similar to the method proposed by Tappen and Liu [14] which was used to solve the face hallucination problem. The image hallucination or superresolution could be treated as an image restoration problem of missing high-frequency components of the original image that need to be restored. However, an image decomposition problem is more ill conditioned and more difficult than face hallucination and image superresolution. The method of Tappen and Liu cannot be directly applied to the task of decomposition of chest radiographs. We need to establish the decomposition model, develop the efficient selection strategy of exemplars and dense matching algorithm for large images, and design the efficient optimization algorithm for the energy function of a decomposition model.

### 2.3. Bayesian Framework for Decomposition of Chest Radiographs

We expressed the decomposition of chest radiographs in a Bayesian MAP inference framework. Given a chest radiograph **Y**, the goal was to find a soft tissue image **S**∗ and a bone image **B**∗ which maximize the posterior:

(1) $$\begin{array}{c} \left\{S*,B*\right\}=\underset{S,B}{\arg\max}p\left(S,B\;|\;Y\right)=\underset{S,B}{\arg\max}p\left(Y\;|\;S,B\right)p\left(S,B\right). \end{array}$$

Assuming that the bone image **B** and the soft tissue image **S** were independent, then

(2) $$\begin{array}{c} \left\{S*,B*\right\}=\underset{S,B}{\arg\max}p\left(Y\;|\;S,B\right)p\left(S\right)p\left(B\right), \end{array}$$

where *p*(**S**) and *p*(**B**) were the probability density functions (image priors) of the soft tissue and bone components, respectively. The likelihood function *p*(**Y** | **S**, **B**) expressed the compatibility between the observed chest radiograph and the decomposed soft tissue and bone components. Considering that we expected a chest radiograph **Y** to be decomposed as **Y** = **S** + **B**, *p*(**Y** | **S**, **B**) is expressed as

(3) $$\begin{array}{c} p\left(Y\;|\;S,B\right)=\frac{1}{Z_{d}}\exp\left(-\lambda {\left\|Y-S-B\right\|}_{2}^{2}\right), \end{array}$$

where *λ* is a tuning coefficient and *Z*_*d*_ is the normalization constant to make *p*(**Y** | **S**, **B**) a valid distribution.

### 2.4. Exemplar-Based Nonparametric Image Priors

The key for successfully decomposing chest radiographs is the effective image priors *p*(**B**) and *p*(**S**). As previously discussed, the general image priors cannot work well in separating the soft tissue component from the bone component. We formed the nonparametric priors from the database of actual DES radiographs. The image triplets in the database were denoted as the set {(**Y**_*i*_, **S**_*i*_, **B**_*i*_), *i* = 1, 2, ⋯, *N*}. The probability density of a soft tissue image **S** in a form of kernel density estimation can be written as

(4) $$\begin{array}{c} p\left(S\right)=\frac{1}{Z_{S}}\overset{N}{\underset{i=1}{\sum }}K\left(S,S_{i};T_{i}\right), \end{array}$$

where *K*(**S**, **S**_*i*_; **T**_*i*_) is a kernel function measuring the similarity between **S** and **S**_*i*_, *Z*_*S*_ is the normalization factor, and **T**_*i*_ is a corresponding field which represents the pixel-to-pixel corresponding between **S** and **S**_*i*_. Using the corresponding field **T**_*i*_, the relationships of image patches were established to align **S** and **S**_*i*_. For example, an image patch centered at *x* in **S** (denoted as *R*_*x*_**S**) corresponded to the image patch centered at *x* + **T**_*i*_(*x*) in **S**_*i*_ (denoted as *R*_*x*+**T***i*(*x*)_ **S**_*i*_).

If the Gaussian kernel is adopted as the kernel function for density estimation, the prior *p*(**S**) can be formulated as

(5) $$\begin{array}{c} p\left(S\right)=\frac{1}{Z_{S}}\overset{N}{\underset{i=1}{\sum }}\exp\left(-\eta_{S}\underset{x\in S}{\sum }{\left\|R_{x}S-R_{x+T_{i}\left(x\right)}S_{i}\right\|}_{2}^{2}\right), \end{array}$$

where *η*_*S*_ is a hyperparameter. Similarly, the prior *p*(**B**) can be written as

(6) $$\begin{array}{c} p\left(B\right)=\frac{1}{Z_{B}}\overset{N}{\underset{i=1}{\sum }}\exp\left(-\eta_{B}\underset{x\in B}{\sum }{\left\|R_{x}B-R_{x+T_{i}\left(x\right)}B_{i}\right\|}_{2}^{2}\right). \end{array}$$

When there are many samples (e.g., *N* is large) in the image database, it would be very expensive (and unnecessary) to construct the priors *p*(**B**) and *p*(**S**) using all samples. To alleviate this problem, a small subset of samples should be selected in the image database as exemplars. The above priors estimated using the selected samples are regarded as exemplar-based priors. The search and selection method for the exemplars and the resolution of corresponding fields will be described in the following subsections.

### 2.5. Preprocessing and Local Feature Descriptors of Chest Radiographs

Due to the differences in acquisition conditions and patients, the density and contrast vary within different chest radiographs, which were acquired by X-ray digital radiography (DR) or computed radiography and DES systems. These differences may affect the comparability of image features. The preprocessing step of contrast normalization is necessary to achieve consistency of chest radiographs. We adopted the guided image filter [18] to enhance the structural details and normalize the contrast of chest radiographs.

A guided image filter is an edge-preserving smoothing filter, which is effective and efficient in many computer vision and graphic applications. The principle of the guided image filter is that the input image is filtered through a guidance image through utilizing the structures in the guidance image. As a result, the output image maintained the overall characteristics and gradients of the input image when the input image is used as the guidance image. For a given chest radiograph **Y**, its smoothed image by the guided image filter with a large radius (e.g., 40 pixels) is used as a base layer **Y**_0_. The detail layer is **Y**_*d*_ = **Y** − **Y**_0_. The chest radiograph **Y** is normalized as

(7) $$\begin{array}{c} \text{Y}⟵{\text{Y}}_{n}=\frac{Y_{d}-\mu_{d}}{\sigma_{d}}, \end{array}$$

where *μ*_*d*_ and *σ*_*d*_ are the intensity mean and standard deviation of **Y**_*d*_, respectively. **Y**_*n*_ is the normalized **Y**. Given that the bone images are rather homogeneous at the large scale, the base layers of the bone images are very homogeneous. Actually, the base layer of **Y** is almost identical to that of the corresponding soft tissue image **S** apart from a global intensity offset. Thus, the soft tissue image **S** is normalized consistently to equation (7) without the loss of structural details as

(8) $$\begin{array}{c} \text{S}⟵S_{n}=\frac{S-Y_{0}-\mu_{S}}{\sigma_{d}}, \end{array}$$

where *μ*_*S*_ is the intensity mean of **S** − **Y**_0_ and **S**_*n*_ is the normalized **S**. And the bone image **B** is normalized as

(9) $$\begin{array}{c} \text{B}⟵B_{n}=\frac{B-\mu_{B}}{\sigma_{d}}, \end{array}$$

where *μ*_*B*_ is the intensity mean of **B** and **B**_*n*_ is the normalized **B**. In this way, the chest radiographs exhibited consistent contrast with the enhanced details, and the relationship **Y** = **S** + **B** between the normalized images was also maintained. The normalized soft tissue/bone images by the use of the proposed normalization procedure can be easily recovered to their original form, and the details of corresponding chest radiographs are enhanced.

In our proposed system, the image representations and the corresponding image patches highly relied on the local feature descriptors. Ideally, the descriptors should have high discriminative power and invariance to image transformations. However, no single kind of dense local descriptor can achieve these two goals very well. We combined three kinds of dense descriptors to describe the local feature and the contextual information of chest radiographs. The first kind of descriptor is the small raw image patch (e.g., 7 × 7 patch). The raw image patches contain the important (normalized) intensity information. The second kind of descriptor exhibits the responses of the modified Leung-Malik (LM) filter bank [19]. The modified LM filter bank consists of the first and second derivatives of Gaussians at six orientations and four scales resulting in a total of 48 filters, one Laplacian of Gaussian filter and one Gaussian filter. The filter scales range from 1 to 32 pixels. The 50-dimensional filter bank responses are normalized by Weber's law, which can obtain the information of small textural and large structures. The third kind of descriptor is the dense SIFT (Scale-Invariant Feature Transform) descriptor [20], which is extracted to characterize local image structures and encode contextual information. For each pixel in an image, its neighborhood (e.g., 16 × 16 block) is divided to a 4 × 4 cell array. The gradient orientations in each cell are quantized into eight bins. The obtained dense SIFT descriptors are 4 × 4 × 8 = 128 dimensional. The combined descriptors are 277 (49 + 50 + 128) dimensional. Finally, we set the different weight factors for the three kinds of descriptor to balance their contributions and reduce the dimensionality of combined descriptors through principal component analysis (PCA) to alleviate computational burden.

### 2.6. Search and Selection of Exemplars from Image Database

Rapid search of similar images for an input image from a database can be performed by comparing the global representations of images. We used BoW image representation [21] as the global representation of the chest radiographs. The BoW image representation is analogous to the BoW representation of text documents, which makes techniques for text retrieval readily applicable to the problem of image retrieval. The BoW model first needs to construct a codebook containing visual words (cluster centers) by clustering invariant descriptors on a given training data set and then exacts the local descriptors of an input image that will be vector quantized with respect to these visual words. Given a codebook, an image is represented as a histogram formed by the number of occurrences of each visual word on the sampled local descriptors from the image. In this study, the codebooks of local descriptors are generated by *k*-means clustering. Since the difference between chest radiographs is subtle, a relatively large codebook is needed. To further improve the descriptive power of BoW histograms, a spatial pyramid model is adopted to incorporate the spatial information of images [22]. Specifically, the spatial pyramid includes two levels: the entire image (level 0) and its four rectangular grid cells (level 1). The BoW histograms of the entire image region and the four subregions are concentrated as the global representation of a chest radiograph.

Let *H*(*k*) denote the *k*_th_ element of a concentrated histogram *H*. The image similarity measure of two images *A* and *B* in the image search stage can be defined as histogram intersection:

(10) $$\begin{array}{c} \text{sim}\left(A,B\right)=\text{sim}\left(H_{A},H_{B}\right)=\underset{k}{\sum }\min\left(H_{A}\left(k\right),H_{B}\left(k\right)\right), \end{array}$$

where the maximum of *k* is 5000. This similarity measure refers to an approximate number of matches between the local descriptors at two spatial levels in the two images. Other histogram similarities or distance such as the Earth Mover's Distance can also be used [23]. The top *M* most similar chest radiographs with the corresponding soft tissue and bone images in the database to a given chest radiograph in terms of similarity measure (equation (10)) are selected as exemplars for the estimation of priors.

### 2.7. Hierarchically Dense Matching of Chest Radiographs

To construct the priors in equations (5) and (6), we determined the dense corresponding fields and matched the pixels between the input and the chest radiographs of selected exemplars. Unlike the traditional dense matching problems such as stereo or nonrigid interpatient registration, in which the two images contain the same scene or objects, we attempted to densely match intrapatient chest radiographs containing different objects with varying appearances and shape. The variations in chest radiographs can make matching of the low-level image patches ambiguous.

To address the dense matching problem, several dense matching methods have been proposed which typically enforce both appearance agreement between matched pixels and geometric smoothness between neighboring pixels, such as SIFT Flow [15] and deformable spatial pyramid (DSP) [16]. SIFT Flow relies on the pixel-level Markov random field (MRF) model with a hierarchical optimization technique. DSP matching uses a pyramid graph model that simultaneously optimizes match consistency ranging from an entire image to coarse grid cells and to every single pixel. Typically, DSP is faster than SIFT Flow because DSP only optimizes the MRF energy in the coarse levels with direct local search in the pixel-level layer. However, DSP uses the downsampled local descriptors in the coarse grid cells that may cause the wrong matching, which cannot be corrected well in the following local search. The PatchMatch algorithm computes fast dense correspondences in another way [24]. For efficiency, this algorithm abandons the global optimization that enforces explicit smoothness on neighboring pixels. Instead, it progressively searches for correspondences by a randomized search technique; a reliable match at one pixel subsequently guides the matching locations of its nearby pixels, thereby implicitly enforcing geometric smoothness. Since the PatchMatch algorithm can only determine a local optimum because of the randomized search and the field propagation strategy, the final correspondence field estimated by PatchMatch highly relies on the initial estimation. The PatchMatch algorithm also discards the prior knowledge on the spatial layout of images, which starts at a totally random initialization.

Matching two images should determine the most similar local feature (match) from one image for each pixel to the other image with the geometric constraint. However, the effective geometric constraints are unclear. Intuitively, the significant matching between chest radiographs should have close appearance and should be located near the same anatomical sites simultaneously. We performed dense matching of chest radiographs in a hierarchical way similar to DSP matching but without the need of energy optimization similar to the PatchMatch algorithm.

**Figure 3:**
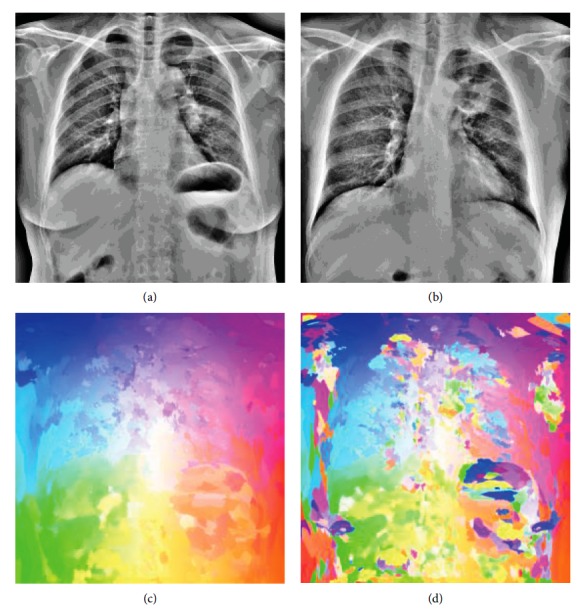
Visualization of corresponding fields.

The input chest radiograph is divided into nonoverlapping rectangular grid cells, and the chest radiographs in the database are divided into overlapping cells with the fixed step size analogous to the DSP matching algorithm. The similarity between grid cells is defined as the intersection of the BoW histogram. The grid cells should be large enough (e.g., 32 × 32 pixels) to estimate the reliable distribution of visual words and identify their anatomical sites. Given that all chest radiographs exhibit a similar spatial layout like that of the clavicles located at the top of the lung field and the hearts located between the left and right lungs, the search for similar grid cells was limited in the local regions of a 1/4 image area. By matching the grid cells, we obtained a very coarse corresponding field **T**. Using **T** with random permutation as the initial estimation of the corresponding field, we applied the field propagation and local randomized search as the PatchMatch algorithm to refine the corresponding field. More details of our hierarchically dense matching are described in Algorithm 1. The corresponding fields of two chest radiographs found by the proposed hierarchically dense matching and PatchMatch algorithms are presented in Figure 3. Obviously, our algorithm can achieve a smoother corresponding field. In contrast, the corresponding field solved by the PatchMatch algorithm lacks consistency due to its overrandomization.

Figure 3 illustrates a visualization of corresponding fields by our proposed hierarchically dense matching and PatchMatch algorithms. Figures 3(a) and 3(b) show two normalized chest radiographs as the source image and target image for dense matching, respectively. Figures 3(c) and 3(d) illustrate the visualization of the corresponding fields solved by our hierarchically dense matching and PatchMatch algorithms, respectively. Corresponding fields in Figures 3(c) and 3(d) are displayed with the same color mapping.

### 2.8. Optimization of Decomposition Energy Function with Exemplar-Based Priors

The MAP estimation of the soft tissue image **S** and the bone image **B** can be rewritten as

(11) $$\begin{array}{c} \left\{S*,B*\right\}=\underset{B,S}{\arg\min}E\left(S,B\right), \end{array}$$

where

(12) $$\begin{array}{c} E\left(S,B\right)=-\log p\left(Y\;|\;S,B\right)-\log p\left(S\right)-\log p\left(B\right)=E_{d}\left(S,B\right)+E_{p}\left(S\right)+E_{p}\left(B\right), \end{array}$$

where *E*_*d*_ and *E*_*p*_ are the data and prior terms, respectively. Ignoring the constant, we obtained

(13) $$\begin{array}{c} E_{d}\left(S,B\right)=\lambda {\left\|Y-S-B\right\|}_{2}^{2}, \end{array}$$

(14) $$\begin{array}{c} E_{p}\left(S\right)=-\log\underset{x}{\sum }\exp\left(-\eta_{S}\overset{M}{\underset{i=1}{\sum }}{\left\|R_{x}S-R_{x+T_{i}\left(x\right)}S_{i}\right\|}_{2}^{2}\right). \end{array}$$

The prior in equation (14) can be considered induced from the density estimated by image-level samplings. As for patch-level samplings, the prior term *E*_*p*_(**S**) can be reformulated as

(15) $$\begin{array}{c} E_{p}\left(S\right)=-\underset{x}{\sum }\log\overset{M}{\underset{i=1}{\sum }}\exp\left(-\eta_{S}{\left\|R_{x}S-R_{x+T_{i}\left(x\right)}S_{i}\right\|}_{2}^{2}\right). \end{array}$$

Compared to equation (14), the form of equation (15) is more flexible, hence adopted in our final decomposition model. Analogously, we can modify the prior term *E*_*p*_(**B**).

The gradients of *E*(**S**, **B**) with respect to **S** and **B** can be derived easily, and the energy function *E*(**S**, **B**) can be minimized by a gradient descent algorithm. However, the gradient descent algorithms usually need many iterations to converge. We proposed an iteratively reweighted least-squares (IRLS) [25] scheme to efficiently minimize the energy function *E*(**S**, **B**) by generating a sequence {**S**_*t*_, **B**_*t*_} via

(16) $$\begin{array}{c} \left\{S^{t+1},B^{t+1}\right\}=\underset{B,S}{\arg\min}\lambda {\left\|Y-S-B\right\|}_{2}^{2}+\eta_{S}\underset{x}{\sum }\overset{M}{\underset{i=1}{\sum }}w_{x,i}^{s}{\left\|R_{x}S-R_{x+T_{i}\left(x\right)}S_{i}\right\|}_{2}^{2}+\eta_{B}\underset{x}{\sum }\overset{M}{\underset{i=1}{\sum }}w_{x,i}^{b}{\left\|R_{x}B-R_{x+T_{i}\left(x\right)}B_{i}\right\|}_{2}^{2}, \end{array}$$

where the weights are

(17) $$\begin{array}{c} w_{x,i}^{s}=\frac{\exp\left(-\eta_{S}{\left\|R_{x}S^{t}-R_{x+T_{i}\left(x\right)}S_{i}\right\|}_{2}^{2}\right)}{\sum_{j=1}^{M}\exp\left(-\eta_{S}{\left\|R_{x}S^{t}-R_{x+T_{j}\left(x\right)}S_{j}\right\|}_{2}^{2}\right)},\\ w_{x,i}^{b}=\frac{\exp\left(-\eta_{B}{\left\|R_{x}B^{t}-R_{x+T_{i}\left(x\right)}B_{i}\right\|}_{2}^{2}\right)}{\sum_{j=1}^{M}\exp\left(-\eta_{B}{\left\|R_{x}B^{t}-R_{x+T_{j}\left(x\right)}B_{j}\right\|}_{2}^{2}\right)}. \end{array}$$

The solution {**S**_*t*+1_, **B**_*t*+1_} can be obtained by solving the following linear equations:

(18) $$\begin{array}{c} \left(\lambda +\eta_{S}\underset{x}{\sum }R_{x}^{T}R_{x}\right)S+\lambda B=\lambda Y+\eta_{S}\overset{M}{\underset{i=1}{\sum }}\underset{x}{\sum }w_{x,i}R_{x}^{T}R_{x+T_{i}\left(x\right)}S_{i},\\ \lambda S+\left(\lambda +\eta_{B}\underset{x}{\sum }R_{x}^{T}R_{x}\right)B=\lambda Y+\eta_{B}\overset{M}{\underset{i=1}{\sum }}\underset{x}{\sum }w_{x,i}^{b}R_{x}^{T}R_{x+T_{i}\left(x\right)}B_{i}. \end{array}$$

Since *w*_*x*,*i*_*R*_*x*_^*T*^ is the operation to rearrange the weighted patches into an image and *R*_*x*_^*T*^*R*_*x*_ is just a diagonal matrix, the linear equations can be easily solved element-wise. The initial solution of **S** and **B** can be obtained by substituting the two prior terms by their quadratic upper bound using Jensen inequality.

### 2.9. Algorithm Summary

The DES image triplets in an established database are denoted as the set {(**Y**_*i*_, **S**_*i*_, **B**_*i*_), *i* = 1, 2, ⋯, *N*}, which were preprocessed and normalized by the use of the approach described in Section 2.5. A PCA projection matrix **P** for local descriptors and a BoW codebook **D** were learned on the samples of local descriptors from the database. For each (normalized) chest radiograph **Y**_*i*_ in the database, the dense local descriptors *F*_*i*_, the spatial pyramid representation *H*_*i*_, and the BoW histograms of the subregions were computed by the use of **P** and **D** in advance.

The proposed decomposition procedure of a new chest radiograph **Y** can be summarized as follows:

**Step 1 .**

Preprocess and normalize the input chest radiograph **Y** according to equation (7). Let **Y**_0_ denote the base layer of **Y**. *μ*_*d*_ and *σ*_*d*_ are the intensity mean and standard deviation of **Y** − **Y**_0_, respectively. The normalized **Y** is computed as Y⟵Y_*n*_ = (**Y**_*d*_ − *μ*_*d*_)/*σ*_*d*_.

**Step 2 .**

Compute the dense local descriptors *F* of **Y** by the use of the PCA projection matrix **P**.

**Step 3 .**

Compute the spatial pyramid representation *H* and the BoW histograms of subregions of **Y**_*n*_ by use of the codebook **D**.

**Step 4 .**

Select the top *M* most similar chest radiographs in the database in terms of similarity measure (equation (10)) as the exemplars of **Y**.

**Step 5 .**

Solve the dense corresponding field **T**_*k*_ between **Y** and **Y**_*k*_ using Algorithm 1 for each exemplar *k* (*k* = 1, 2, ⋯, *M*).

**Step 6 .**

Construct the nonparametric priors for unknown soft tissue image **S** and bone image **B** according to equations (5) and (6).

**Step 7 .**

Optimize the energy function in equation (12) by the use of the IRLS scheme, and solve the soft tissue image **S** and bone image **B**.

**Step 8 .**

Rescale the soft tissue image **S** and bone image **B**, and compensate the base layer of the soft tissue image **S** as **S**⟵*σ*_*d*_**S** + **Y**_0_ + *μ*_*d*_, **B**⟵*σ*_*d*_**B**.

The final decomposition results of the input chest radiograph **Y** are **S** and **B** obtained in Step 8.

### 2.10. Experimental Settings

The experiments were conducted on a PC with a duo Intel Xeon CPU (3.2 GHz) and 16 GB RAM. The implementations were performed using Matlab 2016a with a VLFeat toolbox [26].

The weight coefficients for the three kinds of local descriptors were set to achieve the same variance for each dimension of the combined descriptors. To accelerate the image search and matching procedures, the dimensionality of the combined descriptors was reduced to 60 by PCA, whereas about 98% of variation of the descriptors was maintained. The codebook for BoW representations was generated by *k*-means clustering on the samples of local descriptors from the training data set. The size of each BoW codebook was set to 5000. The codebook was used to compute the BoW histograms for both the image search and hierarchical dense matching. To perform the hierarchically dense matching algorithm, the size *w* of subregion was set to 32 × 32 pixels, and the iteration number of corresponding field propagation and locally randomized search was set to 5. The size of sampling patches for constructing the priors from the actual soft tissue and bone images was set to 5 × 5 pixels.

We used a case-wise procedure to construct the exemplar-based priors and evaluate the performance of the decomposition results. The top *M* most similar cases to the testing chest radiograph among the training set were then selected as the exemplars. The maximum value of *M* was set to 7 in the experiments.

In the energy function of image decomposition, four parameters, namely, *λ*, *η*_*S*_, *η*_*B*_, and the number of exemplars *M*, were considered. *λ* is in the range of [10^−1^ to 10^6^], and *η*_*S*_ is in the range of [10^−6^ to 0.5]. *η*_*B*_ was set as 2 × *η*_*S*_. A large value of *η*_*S*_ would lead to numerical problems. The effect of different parameters was investigated in the following subsections. The average computation time of our decomposition procedure using the unoptimized implementation is 135.8 seconds when the number of selected exemplar images is 5. Most of the computation time is spent in the stage of hierarchically dense matching, and it is dependent on the size of the image and the number of selected exemplars.

The decomposition performance of the soft tissue and bone was quantitatively evaluated using the following measures: The root mean squared error (rmse) is used to evaluate the reconstruction error of the estimated soft tissue/bone image relative to the actual (normalized) soft tissue/bone image, which is defined as

(19) $$\begin{array}{c} \text{rmse}=\sqrt{\frac{1}{n}\underset{x}{\sum }{\left(\hat{Z}\left(x\right)-Z\left(x\right)\right)}^{2}}, \end{array}$$

where $\hat{Z}$ is a reconstructed soft tissue/bone image, *Z* is the corresponding “ground truth” image, *x* denotes the pixel locations in *Z*, and *n* is the number of pixels in the image *Z*. A smaller value of rmse indicates a better estimation of the ground truth. The quality of bone suppression is also evaluated using the bone suppression ratio (bsr) which is defined as [10]

(20) $$\begin{array}{c} \text{bsr}=1-\frac{\sum_{x}{\left(\hat{S}\left(x\right)-S\left(x\right)\right)}^{2}}{\sum_{x}{\left(Y\left(x\right)-S\left(x\right)\right)}^{2}}, \end{array}$$

where $\hat{S}$ is an estimation of the actual soft tissue image **S** and **Y** is the testing chest radiograph. bsr = 1 indicates perfect performance.

If the bone component is treated as a type of structural noise, then the bone suppression procedure of the chest radiograph is considered denoising or filtering. A well-known denoising performance measure called the structural similarity image measure (ssim) [27] can be also used to evaluate the quality of the decomposed soft tissue and bone images. The intensity ranges of images are rescaled into the range of [0 to 255], and the default setting parameters in the implementation (https://ece.uwaterloo.ca/~z70wang/research/ssim/) of ssim are used to compute the values of ssim.

## 3. Experimental Results

### 3.1. Effect of Hyperparameters

**Figure 4:**
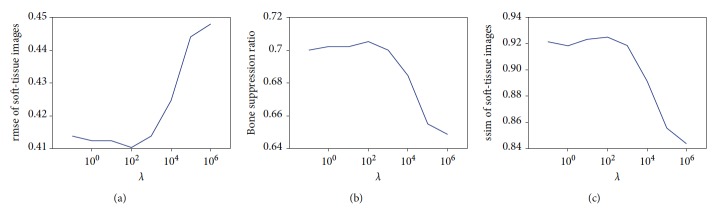
The effect on decomposition performance of parameter *λ* (weight of the data term).

We varied the values of the four parameters *λ*, *η*_*S*_, *η*_*B*_, and *M* to investigate their effect and to determine the proper settings.  Figure 4 shows the average measures of decomposition performance at different *λ* with fixed *η*_*S*_ (*η*_*S*_ = 10^−5^) and fixed *M* (*M* = 5). The exemplar images were selected as described in Subsection 2.5. When the value of *λ* is large, the optimization of the energy function tends to make substantial contributions of the data term to the decomposed images. We observed that larger *λ* leads to better decomposition in terms of three performance measures. However, when the parameter *λ* becomes very large, the decomposition results can be extremely arbitrary and meaningless because of ignoring the use of the prior terms. An appropriate value of *λ* according to the experimental results is 100.

**Figure 5:**
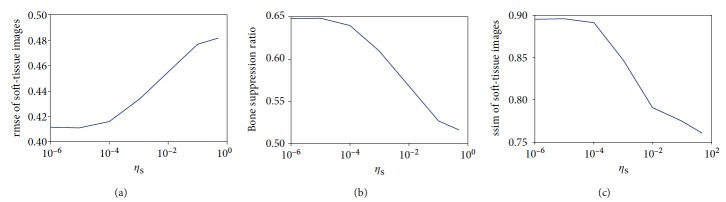
The effect on decomposition performance of parameter *η*_*S*_ for kernel density estimation.

Figure 5 shows the average measures of decomposition performance at different *η*_*S*_ with fixed *λ* (*λ* = 100) and fixed *M* (*M* = 5). From Figure 5, we observed that the lower values of *η*_*S*_ and *η*_*B*_ led to better decomposition. In fact, the optimization of the log-sum-exp function tends to average the matched patches of each position when *η*_*S*_ and *η*_*B*_ have a low value. As the value of *η*_*S*_ and *η*_*B*_ increases, the optimization of the log-sum-exp function more closely approximates the min operation and the decomposed images looks sharper. However, the log-sum-exp functions with the large values of *η*_*S*_ or *η*_*B*_ also introduce artifacts in the decomposed images and results in worse decomposition performance. Based on these results, the parameters *λ* and *η*_*S*_ were set to 100 and 10^−5^ in the subsequent experiments, respectively.

**Figure 6:**
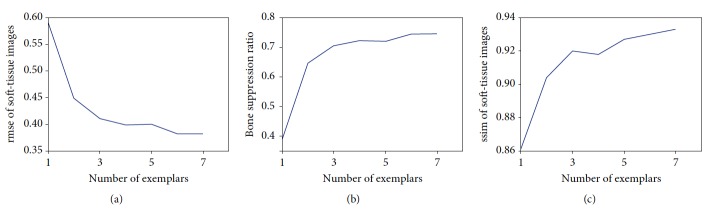
The effect on decomposition performance of the number (*M*).

The number of exemplars *M* is another crucial parameter for decomposition performance.  Figure 6 shows that decomposition performance was improved significantly by increasing the number of exemplars. However, the computation cost of image matching and energy optimization would exponentially increase when many exemplars were used to construct the prior terms. As shown in Figure 6, the improvement in performance is relatively small when the number of exemplars is over 5. The number of exemplars *M* was set to 5 in subsequent experiments if *M* was not specified.

**Figure 7:**
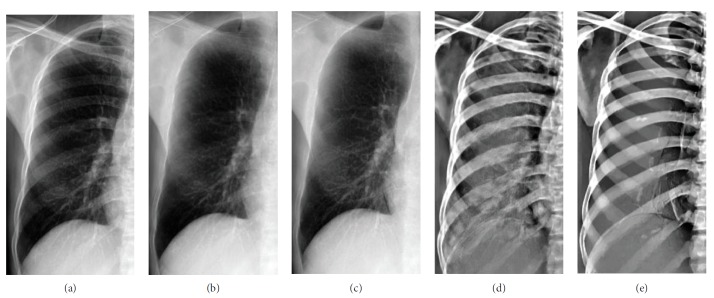
Decomposition results of a chest radiograph without motion artifacts: (a) the right lung field of a chest radiograph; (b) the decomposed soft tissue image; (c) the actual DES soft tissue image; (d) the decomposed bone image; (e) the actual DES bone image.

**Figure 8:**
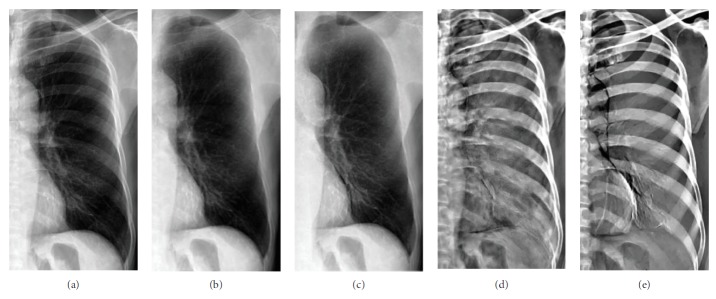
Decomposition results of a chest radiograph with motion artifacts: (a) the left lung field of a chest radiograph; (b) the reconstructed soft tissue image by use of our method; (c) the actual DES soft tissue image; (d) the reconstructed bone image by use of our method; (e) the actual DES bone image with obvious motion artifacts.

**Figure 9:**
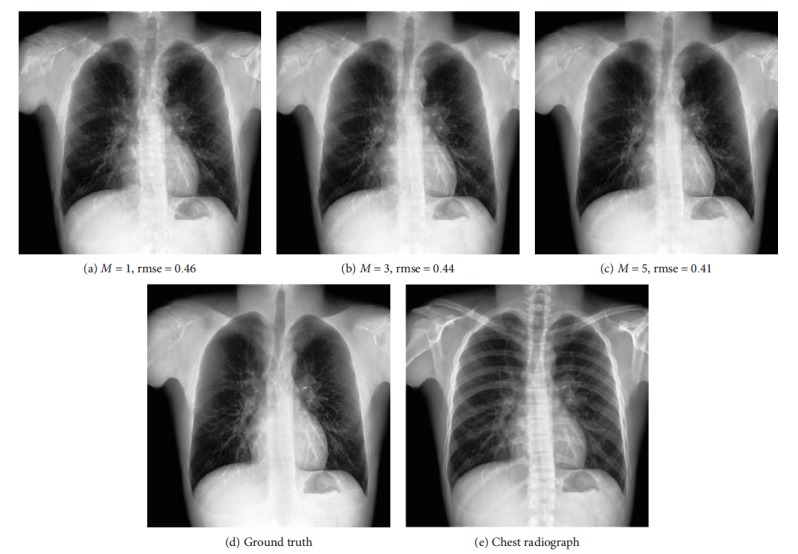
The reconstructed soft tissue images using the different numbers of exemplars. From left to right, the numbers of used exemplars (*M*) are 1, 3, and 5, respectively. rmse is the root mean squared error of the reconstructed soft tissue image. (a–c) are the reconstructed soft tissue images. (d) corresponds to the ground truth. The corresponding standard chest radiograph is (e).

**Figure 10:**
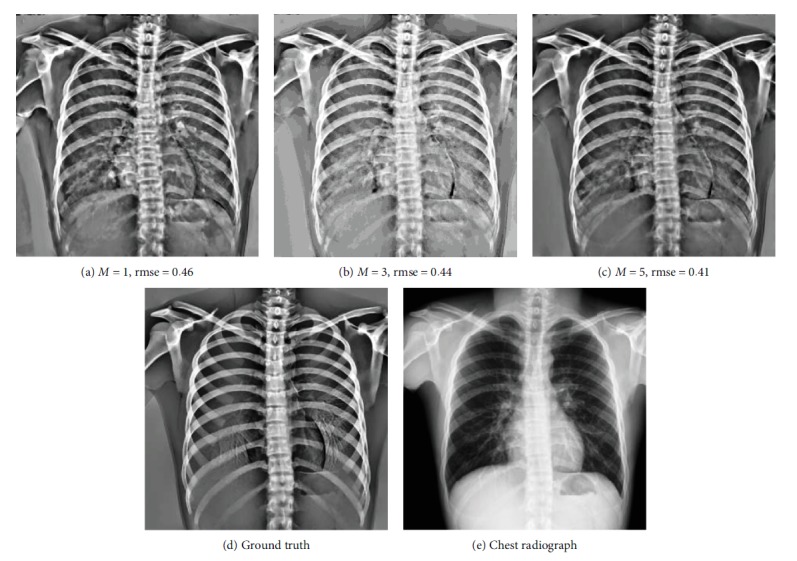
The reconstructed bone images using the different numbers of exemplars. From left to right, the numbers of used exemplars (*M*) are 1, 3, and 5, respectively. rmse is the root mean squared error of the reconstructed soft tissue image. (a–c) are the reconstructed bone images. (d) corresponds to the ground truth. The corresponding standard chest radiograph is shown in (e).

Some examples of decomposition results are illustrated in Figures 7–10. Figures 7 and 8 can be enlarged and viewed on the screen for a better comparison. We observed that the ribs and clavicles are suppressed substantially and the visibility of the soft tissue is maintained in the reconstructed soft tissue images. Visually, the reconstructed soft tissue image and the actual image are similar. Comparing the reconstructed bone images with the actual bone images, some bone edges are more obscure where the bone edges are weak in the observed chest radiograph. In Figure 8, typical motion artifacts in the actual DES bone images were observed. Our decomposition method can reduce the motion artifacts to some extent, as shown in Figure 8(d), because of the smoothing effect of the weighted average of sampling patches. The use of the actual DES soft tissue and bone images with motion artifacts as the ground truth may lead to an overestimated reconstruction error. Figures 9 and 10 show examples of decomposition results using different numbers of exemplars (*M* = 1, 3, and 5). Visual improvement of the estimated soft tissue and bone images is observed when more exemplars are used. Some block artifacts can be observed in the reconstructed soft tissue and bone images using fewer exemplars. These block artifacts were generated because of dissimilar patches in the exemplar chest radiographs for some patches in the input chest radiograph or mismatches between the patches. The selection of similar images as exemplars or using more exemplar images could ensure that each patch in the input chest radiograph has some possible similar patches in the exemplar images and could reduce the block artifacts and reconstruction error. As shown in Figure 9(c), the reconstructed soft tissue image is very similar to the corresponding DES soft tissue image shown in Figure 9(d), and the bone components of the corresponding chest radiograph shown in Figure 9(e) are substantially suppressed. In fact, the ssim index between Figures 9(c) and 9(d) is 0.915. A high ssim index indicates that most of the structures and details of the ground truth image are contained in the reconstructed image. Comparing Figure 9(c) with Figure 9(a), the bone component in Figure 9(c) is suppressed more completely than that in Figure 9(a). The decomposed bone image shown in Figure 10(c) looks clearer compared to that in Figure 10(a), which looks a little messy with fewer exemplars. With more exemplars, the reconstructed bone images show clearer rib edge and are more similar to the DES bone image shown in Figure 10(d).

**Figure 11:**
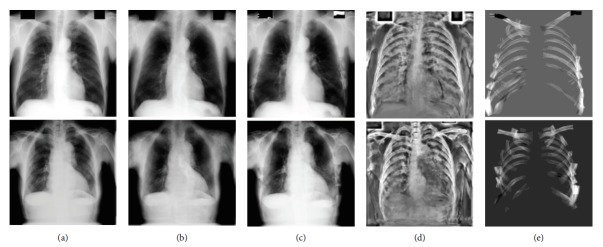
Decomposition results of two chest radiographs from the JSRT data set: (a) the original chest radiographs (“JPCLN063” and “JPCNN071”); (b) the reconstructed soft tissue images by the use of our method; (c) the bone-suppressed images by the use of the gradient modification method; (d) the reconstructed bone images by the use of our method; (e) the estimated bone structures by the use of the gradient modification method.

We used our decomposition method to process the chest radiographs in the JSRT database which is the most commonly used database of chest radiographs for computer-aided detection and processing techniques [28]. Since the corresponding ground truth of the soft tissue and bone images of the JSRT database is unknown, the publicly available bone suppression results provided by Horvath [28] using the gradient modification method were used to be qualitatively compared with the results of our method.  Figure 11 shows the decomposition results of two chest radiographs from the JSRT database. Visually, the reconstructed soft tissue images of our method are more natural. When it is close to the thoracic edge, the soft tissue image reconstructed by the gradient modification method produced the shadows apparently. The two methods had advantages and disadvantages. The results of the gradient modification method depend on the segmentation of ribs and clavicles, which might be insensitive to the types of acquisition equipment of chest radiographs. However, the shadows of bones, which were not segmented, could not be removed. The results of our method depend on the appearance of the chest radiographs. Even if the images in the JSRT database are the scanned films and the number of DES exemplar is limited, our method could work well in most cases.

### 3.2. MAP Decomposition versus Locally Weighted Regression

Compared with the decomposition method that minimized the MAP energy function using the exemplar-based prior term, a more simple and direct method for estimating soft tissue and bone images is the weighted regression, which is analogous to label transfer [29]. Considering the sampling patches {*p*_*i*_, *i* = 1, ⋯, *M*} from the exemplar images based on the corresponding fields as the nearest neighbors, a soft tissue or bone image patch can be estimated by locally weighted regression as $\hat{p}=\sum_{i=1}^{M}w_{i}p_{i}$, where the weight *w*_*i*_ is defined based on the matching error of the local descriptor. The result of locally weighted regression can be considered the minimum mean square estimation of the soft tissue or bone image patch. The entire soft tissue/bone image is reconstructed by rearranging all of the estimated patches. The locally weighted regression method is similar to the kNN regression method proposed by [7]. The main difference is the search method of kNN and the local descriptor used.

The mean and standard deviation of performance measures for MAP decomposition and locally weighted regression are listed in Table 1. From the results shown in Table 1, our method is significantly superior to the locally weighted regression. rmse is computed on the normalized soft tissue and bone images. The MAP decomposition method yields a lower rmse and a higher bone suppression ratio than the locally weighted regression. The values of the ssim of the reconstructed soft tissue images by two methods are 0.927 and 0.846, respectively. The high ssim indicates that the detail structures in the DES soft tissue image are preserved by two decomposition methods. Since the intensity variations of soft tissue images are significantly larger than those of bone images, the ssim of the reconstructed bone image is lower than the ssim of the reconstructed soft tissue images.

**Figure 12:**
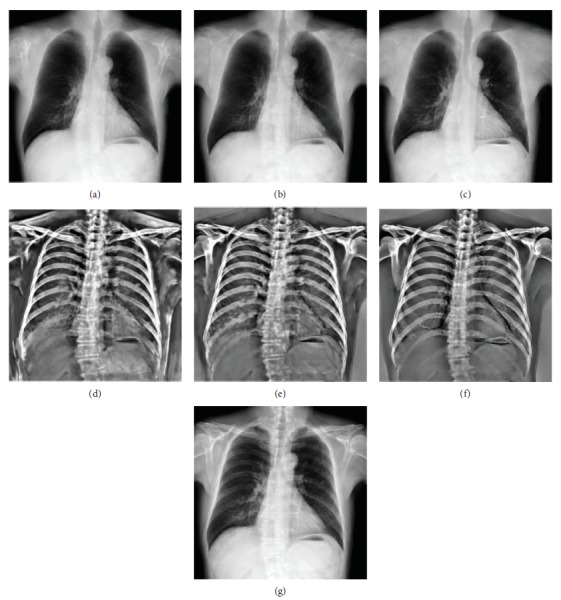
Comparison of the decomposition results through the MAP model and the locally weighted regression method: (a) the reconstructed soft tissue image through the locally weighted method; (b) the decomposed soft tissue image through the MAP model; (c) the DES soft tissue image of (g); (d) the reconstructed bone image through the locally weighted method; (e) the decomposed bone image through the MAP model; (f) the DES bone image of (g); (g) the standard chest radiograph.

The decomposition results of a chest radiograph by the use of the MAP model and the locally weighted regression method are shown in Figure 12. The soft tissue images and bone images reconstructed by the MAP model in Figure 12 are visually much closer to the ground truth than that by the locally weighted regression method. And the reconstruction errors (rmse) of the soft tissue image estimated by the MAP model and the locally weighted regression are 0.41 and 0.44, respectively. Actually, the optimization of the MAP energy function with the data term tends to satisfy the constraint **Y** = **S** + **B** and utilizes more information on the input chest radiograph to reduce the reconstruction error and generate higher fidelity results. By contrast, the locally weighted regression cannot ensure that ‖**Y** − **S** − **B**‖^2^ can be minimized definitely. Thus, the locally weighted regression can only yield worse estimation of the soft tissue and bone images than the MAP decomposition.

## 4. Discussion

In our MAP decomposition model, the prior terms are rendered in the log-sum-exp format. For small values of the parameter *η*_*S*_ or *η*_*B*_, the prior terms can be considered the approximations to the averaging function of quadratic errors between image patches. From the experimental results, we observed that the small values of the parameter *η*_*S*_ or *η*_*B*_ could lead to better decomposition results of the chest radiograph in terms of the three performance measures. The optimal values of *η*_*S*_ and *η*_*B*_ can be dependent on the data set and the performance measures. It is interesting to investigate the other forms of the prior term using other kernel functions for density estimation or the robust loss functions as prior terms for decomposition. Combining the exemplar-based priors, the general image priors, such as total variation [11] and sparsity [30], would be helpful in further improving the decomposition model. Additionally, some methods on image quality improvement can be considered to further enhance the algorithm performance, such as convolution network-based processing [31], fuzzy similarity-based method [32], and sparse coding-based processing [33–35].

The basis of our method is the database of DES radiographs, which is used to estimate image priors. In theory, the estimation accuracy of image (patch) prior probability depends on the samples. However, even the soft tissue and bone components cannot be separated perfectly through using a DES system. Furthermore, a few motion artifacts are present in the soft tissue and bone images of two-exposure DES as a result of cardiac motion and breath. The soft tissue and bone components were also not successfully separated in the regions with motion artifacts. In this work, we acquired enough DES radiographs from Nanfang Hospital, Guangzhou, China, which is useful for that similar patches, for a patch in the source chest image can be found in the selected exemplars of the training set more possibly. From the experimental results, larger *λ* (weight of the data term) can lead to better decomposition in terms of the three performance measures. The data term had substantial contributions to decomposition performance, and the MAP decomposition model was effective for the separation of bone images from the chest radiographs. But this does not indicate that the prior terms are not helpful for decomposition since the decomposition results can be extremely arbitrary and meaningless without the prior terms. Actually, the decomposed soft tissue image **S** and bone image **B** only tend to satisfy the constraint **Y** = **S** + **B** when the parameter *λ* becomes very large.

One bottleneck of our method is the large computation cost mainly because of dense matching between chest radiographs. Although the local descriptors and BoW histograms of the images in the database have been computed off-line and restored, the running time of our method (135.8 s per image of 512 × 512 pixels) is still longer than that of MTANN regression (1.63 s per image of 512 × 512 pixels). Actually, with the popularity of convolutional neural networks (CNN), we also proposed a cascade architecture of CNN (called CamsNet) [36] to improve the results of our MAP model and reach a better result.

The ultimate goal of decomposition or bone suppression of chest radiographs is to improve the performance of radiologists in diagnosing lung diseases. But this ultimate goal cannot be realized directly. Considering that the usefulness of DES soft tissue images had been proved, our decomposition method is aimed at producing the decomposition results similar to the DES soft tissue and bone images as possible. A very small reconstruction error (e.g., rmse) and a very high bone suppression ratio may indicate indirectly the useful decomposition results. However, preserving the details in the abnormal regions and enhancing the contrast of the nodules are important. The data (fidelity) term in the MAP decomposition model can provide a trade-off to balance structure preserving and smoothing. It would be helpful to integrate some general image priors, such as total variation, sparsity, or low rank for bone images with the MAP model. The decomposed bone image would be smoother, and more details of the input chest radiograph would be maintained in the decomposed soft tissue image. The detectability of nodules in the decomposed soft tissue images can be further improved by designing the decomposition energy functions using a certain probability of abnormality detection or optimizing the local descriptors for reducing the mismatches of image patches in abnormal regions. And a more specific nodule detection algorithm should be also developed for the decomposed soft tissue images. Furthermore, the usefulness of our decomposition results for improving the performance of radiologists in diagnosing lung diseases will be investigated in the future.

## 5. Conclusions

We presented a decomposition method of chest radiographs using the exemplar-based nonparametric priors of soft tissue and bone images. Using the real DES radiographs as the exemplars of a chest radiograph for decomposition, the nonparametric priors of the soft tissue and bone images were estimated on the samples of image patches, which were sampled based on dense matching of chest radiographs. Integrating the nonparametric priors into a MAP model, the soft tissue and bone images were reconstructed by minimizing the energy function with the proposed efficient optimization algorithm. Our method could produce soft tissue and bone images like the real DES system but only needed a single conventional chest radiograph as the input. Experiments on synthetic DES radiography and the JSRT database showed that our method could be used to suppress the bone structures in the chest radiographs, which would be potentially useful for radiologists to diagnose lung diseases in chest radiographs.

## Figures and Tables

**Algorithm 1:**
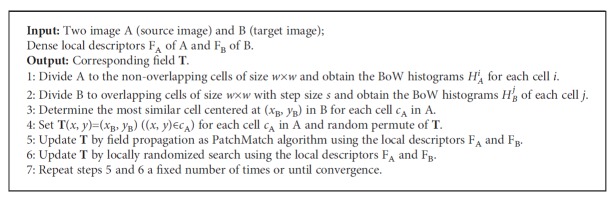
Hierarchically dense matching.

**Table 1:** The average performance of MAP decomposition and locally weighted regression on the validation set with 100 standard chest radiographs. bsr denotes the bone suppression ratio. rmse-S and ssim-S denote the root mean squared error (rmse) and the structural similarity image measure (ssim) for the reconstructed soft tissue, respectively.

Method	bsr	rmse-S	ssim-S
MAP decomposition	0.715	0.414	0.927
Weighted regression	0.704	0.441	0.846

## Data Availability

The image data used in this study were collected from two data sets. The first data set consisted of posterior-anterior DES chest radiographs acquired with a DES system (Revolution XR/d, GE) at Nanfang Hospital, Guangzhou, China, and so cannot be made freely available. The second one was the publicly available Japanese Society of Radiological Technology (JSRT) data set.
